# Detection of antimicrobial traits in fluorescent pseudomonads and molecular characterization of an antibiotic pyoluteorin

**DOI:** 10.1007/s13205-016-0538-z

**Published:** 2016-10-20

**Authors:** J. U. Vinay, M. K. Naik, R. Rangeshwaran, G. Chennappa, Sohel S. Shaikh, R. Z. Sayyed

**Affiliations:** 1 Department of Plant Pathology, University of Agricultural Science, Raichur, India; 2 Department of Microbiology, NBAIR, Bangalore, India; 3 Department of Microbiology, PSGVP Mandal’s, Shri S I Patil Arts, G B Patel Science and STSKVS Commerce College, Shahada Dist, Nandurbar, MS 425 409 India

**Keywords:** Antibiotic, Fluorescent pseudomonads, Pyoluteorin, TLC, TOF–MS

## Abstract

Thirty isolates of fluorescent pseudomonads were obtained from rhizosphere of different crops in Raichur, India. The fluorescent pseudomonad strains were characterized in vitro for biochemical traits, antimicrobial traits, and pyoluteorin antibiotic production. All the isolates that showed fluorescent pigment production under UV light were rod shaped, Gram negative, positive for oxidase, catalase and citrate utilization tests, and negative for indole test. Out of 30 isolates, 07 isolates were positive for HCN production, 15 isolates were positive for H_2_S production, and all the isolates were positive for siderophore production. Among all the isolates, RFP-22 showed the maximum percent inhibition of mycelium (46.66 %) of *Rhizoctonia solani*, the pathogen, and the remaining isolates showed the moderate to least inhibition of mycelium growth of *R. solani*. The 16S rRNA analysis confirmed that the antibiotic positive isolates belonged to genus *Pseudomonas.* The amplification of 779 bp region in isolates RFP- 4 and RFP-19 corresponded to pyoluteorin antibiotic-coding *pltB* gene. Further characterization of pyoluteorin antibiotic through TLC and TOF–MS analysis confirmed the presence of pyoluteorin at 274.26 (g/mol) peak and 2.10 min retention time. Biochemical and molecular analyses confirmed the antagonism of *Pseudomonas* and isolate through pyoluteorin production.

## Introduction

Some of the rhizobacteria positively influence on plant growth and health which were referred as Plant Growth Promoting Rhizobacteria (PGPR). These rhizobacteria are abundant in rhizosheric soil of several crops, and these maintained the ecological balance in niche needed for their survival. These PGPR played a pivotal role in both growth promotion and plant disease control. The mechanisms of growth promotion by these PGPRs are complex and appear to comprise both changes in the microbial balance in the rhizosphere and alterations in host plant physiology (Bashan and Bashan 2005). Biological control of diseases by plant growth promoting rhizobacteria was well-established phenomenon (Shaikh and Sayyed 2015; Shaikh et al. 2016). The antibiotics, HCN, H_2_S, and siderophore have been shown to play a major role in the suppression of several plant pathogens (Handelsman and Stabb 1996; Shaikh et al. 2014).

Among rhizobacteria, *Pseudomonas* sp. are often used as model root-colonizing bacteria (Lugtenberg et al. 2001). The plant growth promoting strain fluorescent *Pseudomonas* are known to produce an array of antibiotics, such as 2,4-diacetylphloroglucinol (2,4-DAPG), pyoluteorin (PLT), pyrrolnitrin (PRN), phenazine-1-carboxyclic acid (PCA), 2-hydroxyphenazines, phenazine-1-carboxamide (PCN), rhamnolipids, oomycin A, cepaciamide A, ecomycins, viscosinamide, and karalicin which are capable of suppressing a broad spectrum of plant pathogens. Among them pyoluteorin, 2,4-DAPG and phenazine are major determinants in biocontrol activity (Borauh and Kumar 2002).

Pyoluteorin was aromatic phenolic polyketide antibiotic that was first isolated from *P. aeruginosa* (Takeda 1958) and later from *P. fluorescens* strains Pf-5 and CHA0 (Bencini et al. 1983). PLT has bactericidal, herbicidal, and fungicidal activities, in particular against *Pythium* spp. (Takeda 1958). Howell and Stipanovic (1980) first established the importance of pyoluteorin antibiotic production in suppression of seedling pathogens, such as *Pythium ultimum*, causing damping off in cotton using *P. fluorescens* pf-5. Similarly, the efficacy of *P. fluorescens* (Pf-5) could be improved by altering the expression of the pyoluteorin producing genes in such a way that effective concentrations of the antibiotic are reached in the seed spermosphere more quickly after a seed was planted (Kraus and Loper 1995). Genetic analysis of several *Pseudomonas* strains has also established a positive correlation between antibiotic production and disease suppression (Homma and Suzui 1989).

The present investigation is aimed at screening of pyoluteorin gene and antimicrobial traits in several fluorescent pseudomonad strains of different crop rhizospheres through PCR and to characterize pyoluteorin antibiotic through thin-layer chromatography and Time-of-Flight–Mass spectroscopy (TOF–MS).

## Materials and methods

### Isolation and biochemical characterization of fluorescent *Pseudomonas* isolates

Isolation of fluorescent *Pseudomonas* was carried out by serial dilution technique. The 0.1 ml of 10^−5^ and 10^−6^ was placed on King’s B medium to isolate the colonies. Plates were incubated at 28 °C for 48 h. After incubation, well-separated individual colonies with yellow green and blue white pigments were marked and detected by viewing under UV light.

Gram staining and pigment production in different isolates of fluorescent *Pseudomonas* was carried out according to the Laboratory Guide for Identification of Plant Pathogenic Bacteria published by the American Phytopathological Society (Schaad 1992).

Simmon’s citrate agar medium was adjusted to pH 7.0, and then, the slants were prepared. Culture was streaked in the slant. The citrate utilization positive reaction was indicated by the change in the colour of the media from green to Prussian blue (Simmons 1926).

Oxidase, catalase, and indole tests were also carried out as per the protocol given by Vanitha et al. (2009). The oxidase and catalase tests were carried out to confirm the aerobic nature of pseudomonads. In oxidase test, a culture grown for 24 h in NA supplemented with 1 % glucose was used. Loopful cells were rubbed onto a filter paper impregnated with 1 % (w/v) aqueous tetra-methyl-p-phenylene diamine dihydrochloride solution. A change in the colour of the cultures to deep purple within 10 s was registered as a positive result (Kovacs 1956). In catalase test, a loopful of 48 h growth of the test bacterium was smeared on a slide and was covered with few drops of hydrogen peroxide. The reaction was noticed as positive if gas bubbles were produced.

For indole test, tryptone broth was inoculated with a small amount of a pure culture of *Pseudomonas* sp. and incubated at 35 °C for 48 h. Five drops of Kovac’s reagent were added directly to the tubes to test the indole production. The reaction was noticed as positive if there is a formation of a pink-to-red colour in the reagent layer on top of the medium.

### Characterization of antimicrobial traits in fluorescent pseudomonads

#### Hydrogen sulfide test

Bacterial isolates were stab inoculated to test tube containing the SIM agar medium and were incubated at 37 °C for 48 h. Black colouration along the line of stab inoculation indicated positive reaction (Vanitha et al. 2009).

#### Hydrogen cyanide production

A strip of sterilized filter paper saturated with a solution containing picric acid, 0.5 %, and sodium carbonate (2.0 %) was placed inside the NA slants amended with glycine (4.4 g/l) and FeCl_3_·6H_2_O (0.3 mM) which were already inoculated with test bacteria and hermetic sealing was done. Change in colour of the filter paper from yellow to brown in 5 days was considered as positive reaction (Bakker and Schippers 1987).

#### Siderophore production

Siderophore production was determined by Chrome Azurol S (CAS) assay. The CAS agar medium was prepared according to procedure given by Schwayn and Neilands (1987). The bacterial culture streaked on the CAS plate. Change of medium colour to orange or presence of light orange halo surrounding the bacterial growth indicates siderophore production by the bacterial isolates.

### Screening of fluorescent pseudomonads for testing bioefficacy against *Rhizoctonia solani* by dual culture

The pseudomonads were streaked 1 day earlier to the test pathogen. They were incubated for control reaches periphery of plates. The diameter of the colony of the fungus was measured in both directions and average was recorded and the percent inhibition on growth of the test fungus was calculated using the formula given below by (Vincent 1927):

$$I = \frac{C - T}{C} \times 100$$

where *I* = Percent inhibition, *C* = Radial growth of fungus in control, and *T* = Radial growth of fungus in treatment.

### Molecular characterization of pyoluteorin gene

#### Genomic DNA isolation

The selected isolates were inoculated in the LB broth and kept overnight for incubation in a shaker at 150 rpm. The DNA was isolated from the selected isolates following procedure using HiPurA™ Bacterial and Yeast Genomic DNA Purification Spin Kit HIMEDIA.

#### Molecular characterization of pyoluteorin gene through PCR

The PCR reaction (25 μL) consisted of 50 ng of genomic DNA, 1× Taq DNA polymerase buffer, 0.5 U of *Taq* DNA polymerase, 0.2 mM of each dNTP, 1.5 mM MgCl_2,_ and 50 mol of primers pltBf (CGGAGCATGGACCCCCAGC) and pltBr (GTGCCCGATATTGGT CTTGACCGAG). Amplification was performed in a DNA thermal cycler with the initial denaturation at 94 °C for 3 min, 30 cycles of 94 °C for 60 s, 58 °C for 45 s, and 72 °C for 60 s and final extension at 72 °C for 10 min. Then aliquots of 10–15 μL of each amplification product was electrophoresed on 0.8 % agarose gel in 1× Tris–acetate–EDTA (TAE) buffer (40 mM Tris-acetate, 1 mM EDTA, pH 8.0) at 50 V for 1–3 h and stained with ethidium bromide and the PCR products were visualized under a UV transilluminator. Following this, 100 bp ladder was used as size markers.

#### Amplification and sequencing of 16S rRNA gene

Amplification of 16S rRNA gene from the genomic DNA of bacteria was carried out using universal primer set, forward primer fD1 (AGTTTGATCCTGGCTCA), and reverse primer rP2 (ACGGCTACCTTGTTACGACTT). PCR cocktail (50 μl) contained 50 pM of primer, 50 ng of genomic DNA, 1X *Taq*DNA polymerase buffer, 1 U of *Taq*DNA polymerase, 0.2 mM of each dNTPs, and 1.5 mM MgCl_2_. The thermal cycling program for the amplification of 16S rRNA gene consisted of initial denaturation at 94 °C for 1 min, 30 cycles of denaturation at 94 °C for 1 min, annealing at 46 °C for 30 s, and extension at 72 °C for 4 min with a final extension at 72 °C for 10 min. Amplification was performed in a DNA thermal cycler. A 5-μl aliquot of each amplification product was electrophoresed on a 1.5 % agarose gel in 1× TAE buffer at 50 V for 45 min, stained with ethidium bromide and the PCR products were visualized with a UV transilluminator.

### Detection of pyoluteorin antibiotic through thin-layer chromatography (TLC)

The pyoluteorin antibiotic production by strain of *P. putida* was determined using a modified method of Chang and Blackwood (1969). Bacterial culture was grown in 5 ml of pigment production medium (peptone, 20 g; glycerol, 20 ml; NaCl, 5 g; KNO_3_, 1 g; distilled water, 1 l; pH 7.2) for 4 days on a rotary shaker at room temperature (28 ± 2 °C). The culture was centrifuged at 3500 rpm for 5 min; the supernatant collected, acidified to pH 2 with 1 N HCl, and then extracted with an equal volume of ethyl acetate. The ethyl acetate extract was reduced to dryness in vacuo and the residue dissolved in methanol. Twenty microlitre samples was applied to thin-layer chromatography plates coated with a 250 μm layer of silica gel and developed in chloroform and methanol (9:1, *v*/*v*) and benzene and acetic acid (9.5:0.5, *v*/*v*) for pyoluteorin as solvent system. The spots were visualized by spraying with diazotized sulphanilic acid or under UV at 254 nm. *Rf* values of the spots were compared with synthetic antibiotics.

### Detection of pyoluteorin antibiotic through time-of-flight–mass spectroscopy

The dried and purified antifungal metabolite was collected and crystallized by dissolving in ethyl acetate (6 ml) and filtered to remove insoluble impurities. The filter was washed with ethyl acetate (2 ml), and the wash was added to ethyl acetate solution. After leaving this solution over night at −20 °C, the crystals were collected. The crystallized antifungal metabolite was re-suspended in a minimum quantity of acetone and analyzed by mass spectroscopy and it was used to identify the retention time and molecular weight of antibiotic.

## Results

### Isolation and biochemical characterization of fluorescent *Pseudomonas* isolates

Thirty fluorescent *Pseudomonas* isolates were collected from the rhizosphere soil of ground nut, pigeon pea, chick pea, sesame, tomato, chilli, sunflower, castor, maize, rice, ragi, niger, safflower, cowpea, horse gram, chrysanthemum, guar, soybean, *Vinca rosea,* and marigold from the farm of University of Agricultural Sciences, Raichur campus, and surrounding villages. In addition, some samples were also collected from Dharwad and Chitradurga. Isolation was done using King’B media by the serial dilution method.

All the isolates showed Gram-negative reaction and rod shape, and pigment production was seen in both *Pseudomonas* agar F medium and fluorescence under UV light that confirmed that all the isolates belong to fluorescent pseudomonads group. Further characterization of them revealed that all the isolates were positive for oxidase test, catalase test, and citrate utilization and were negative for indole test.

### Antimicrobial traits of fluorescent *Pseudomonas* isolates

The fluorescent *Pseudomonas* isolates are known to produce many secondary metabolites, such as hydrogen cyanide (HCN), hydrogen sulfide (H_2_S), and siderophores which are antagonistic properties against many phytopathogens. Among 30 isolates, seven isolates RFP-3, RFP-5, RFP-14, RFP-19, RFP-20, RFP-21, and RFP-26 were positive for HCN production and 15 isolates were positive for H_2_S production (Table 1).

**Table 1:** Characterization of fluorescent pseudomonads isolates for antimicrobial traits

Sl. no.	Isolates	H_2_S production	HCN production	Siderophore*
1	RFP-1	Positive	Negative	Medium
2	RFP-2	Negative	Negative	Medium
3	RFP-3	Positive	Positive	Poor
4	RFP-4	Positive	Negative	Poor
5	RFP-5	Negative	Positive	Strong
6	RFP-6	Positive	Negative	Medium
7	RFP-7	Positive	Negative	Strong
8	RFP-8	Negative	Negative	Poor
9	RFP-9	Positive	Negative	Poor
10	RFP-10	Positive	Negative	Poor
11	RFP-11	Negative	Negative	Poor
12	RFP-12	Positive	Negative	Poor
13	RFP-13	Positive	Negative	Poor
14	RFP-14	Positive	Positive	Poor
15	RFP-15	Negative	Negative	Strong
16	RFP-16	Negative	Negative	Medium
17	RFP-17	Negative	Negative	Poor
18	RFP-18	Negative	Negative	Strong
19	RFP-19	Positive	Positive	Medium
20	RFP-20	Negative	Positive	Poor
21	RFP-21	Positive	Positive	Medium
22	RFP-22	Negative	Negative	Medium
23	RFP-23	Negative	Negative	Poor
24	RFP-24	Positive	Negative	Strong
25	RFP-25	Negative	Negative	Poor
26	RFP-26	Positive	Positive	Medium
27	RFP-27	Negative	Negative	Poor
28	RFP-28	Positive	Negative	Strong
29	RFP-29	Negative	Negative	Poor
30	RFP-30	Negative	Negative	Poor

* Orange colour zone, strong >4 mm, medium 2–4 mm, poor <2 mm

All the isolates of fluorescent *Pseudomonas* sp. were shown positive for siderophore production on CAS agar medium which was indicated by production of yellow/orange-coloured zone surrounding the bacterial growth. Among 30 isolates, six isolates produced higher siderophore production (>4 mm orange colour zone), eight isolates produced moderate siderophore production (2–4 mm orange colour zone), and 16 isolates produced less siderophore production (<2 mm orange colour zone) (Table 1).

### Screening of fluorescent pseudomonads for testing the bioefficacy against *Rhizoctonia solani* by dual culture

Efficacy of 30 indigenous fluorescent pseudomonads was studied under in vitro by screening against *Rhizoctonia solani* using the dual culture method. The results on inhibition of mycelial growth of *R. solani* was recorded and presented here under. Among the isolates tested, RFP-22 showed the maximum percent inhibition of mycelium (46.66 %) and the second highest percent inhibition by RFP-6 (45.55 %), followed by RFP-19 (44 %), RFP-3 (44 %), RFP-21 (43.33 %), and RFP-7 (42.22 %). The remaining isolates showed the moderate inhibition *R. solani*. The RFP-9 (16.66 %) and RFP-17 (6.66 %) showed least inhibition of pathogen (Table 2).

**Table 2:** In vitro efficacy of fluorescent pseudomonads isolates against *R. solani,* the causal agent of sheath blight of rice

Sl. no.	Isolates	Percent inhibition
1	RFP-1	37.22 (37.43)
2	RFP-2	35.00 (36.08)
3	RFP-3	44.00 (41.37)
4	RFP-4	37.77 (37.56)
5	RFP-5	37.77 (37.56)
6	RFP-6	45.55 (42.17)
7	RFP-7	42.22 (40.34)
8	RFP-8	36.66 (36.99)
9	RFP-9	16.66 (23.65)
10	RFP-10	41.11 (39.67)
11	RFP-11	38.88 (38.26)
12	RFP-12	38.88 (38.26)
13	RFP-13	34.44 (35.24)
14	RFP-14	36.66 (36.99)
15	RFP-15	33.33 (35.05)
16	RFP-16	35.00 (36.10)
17	RFP-17	6.66 (14.43)
18	RFP-18	37.77 (37.66)
19	RFP-19	44.00 (41.37)
20	RFP-20	41.11 (39.67)
21	RFP-21	43.33 (41.00)
22	RFP-22	46.66 (42.83)
23	RFP-23	21.11 (27.24)
24	RFP-24	30.00 (33.04)
25	RFP-25	40.00 (39.11)
26	RFP-26	41.66 (39.86)
27	RFP-27	35.55 (36.25)
28	RFP-28	32.22 (34.36)
29	RFP-29	32.77 (34.63)
30	RFP-30	28.66 (33.17)
31	Control	0.00
	SEM±	0.52
	CD (*P* = 0.01)	2.03

* Figures in the parentheses are arc sine values

### Molecular characterization of pyoluteorin gene

**Fig. 1:**
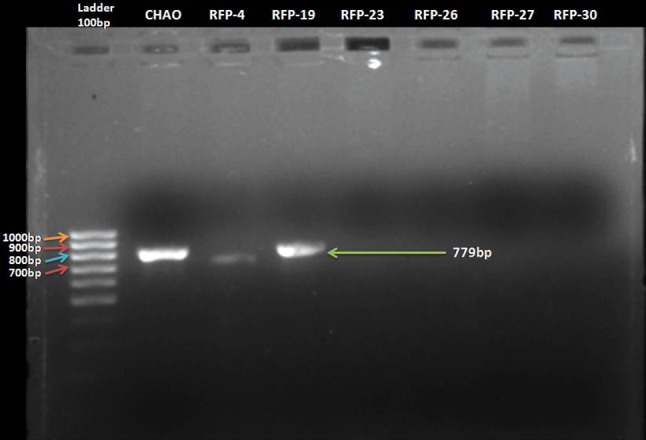
Molecular detection of pyoluteorin (pltB) antibiotic gene in RFP-4 and RFP-19 strains of fluorescent pseudomonads

The fluorescent pseudomonads isolates were screened for pyoluteorin antibiotic gene through PCR using gene specific primers pltC1, pltC2, pltBf, and pltBr. The isolates, namely, RFP- 4 and RFP-19, showed amplification of 779 bp region which corresponded to pyoluteorin antibiotic-coding *pltB* gene (Fig. 1). The remaining isolates did not show any amplification in this region of genome.

### The 16S rRNA amplification of *pltB* gene positive of fluorescent pseudomonads isolates

The extracted DNA of pyoluteorin positive isolates (RFP-4 and RFP-19) were amplified with 16S rRNA universal primers fD1 and rP2. The two fluorescent pseudomonads isolates were amplified and amplified product were checked on 1.5 % agarose gel. The size of amplified DNA showed 1500 bp in length. Sequencing and BLAST of them in NCBI revealed that both the isolates belonged to *Pseudomonas putida.*

### Detection of pyoluteorin antibiotic through TLC

The antibiotic production was tested by growing the antibiotic positive strains in pigment producing medium and extracted with ethyl acetate. The production of pyoluteorin antibiotic in RFP-4 and RFP-19 strains was confirmed by TLC at *Rf* value 0.50 in TLC plate in chloroform: acetone (9:1 *v*/*v*) solvent system. This was compared with the standard reference antibiotic which was co-migrated with sample in TLC plate.

### Detection of pyoluteorin through time-of-flight–mass spectroscopy (TOF–MS)

**Fig. 2:**
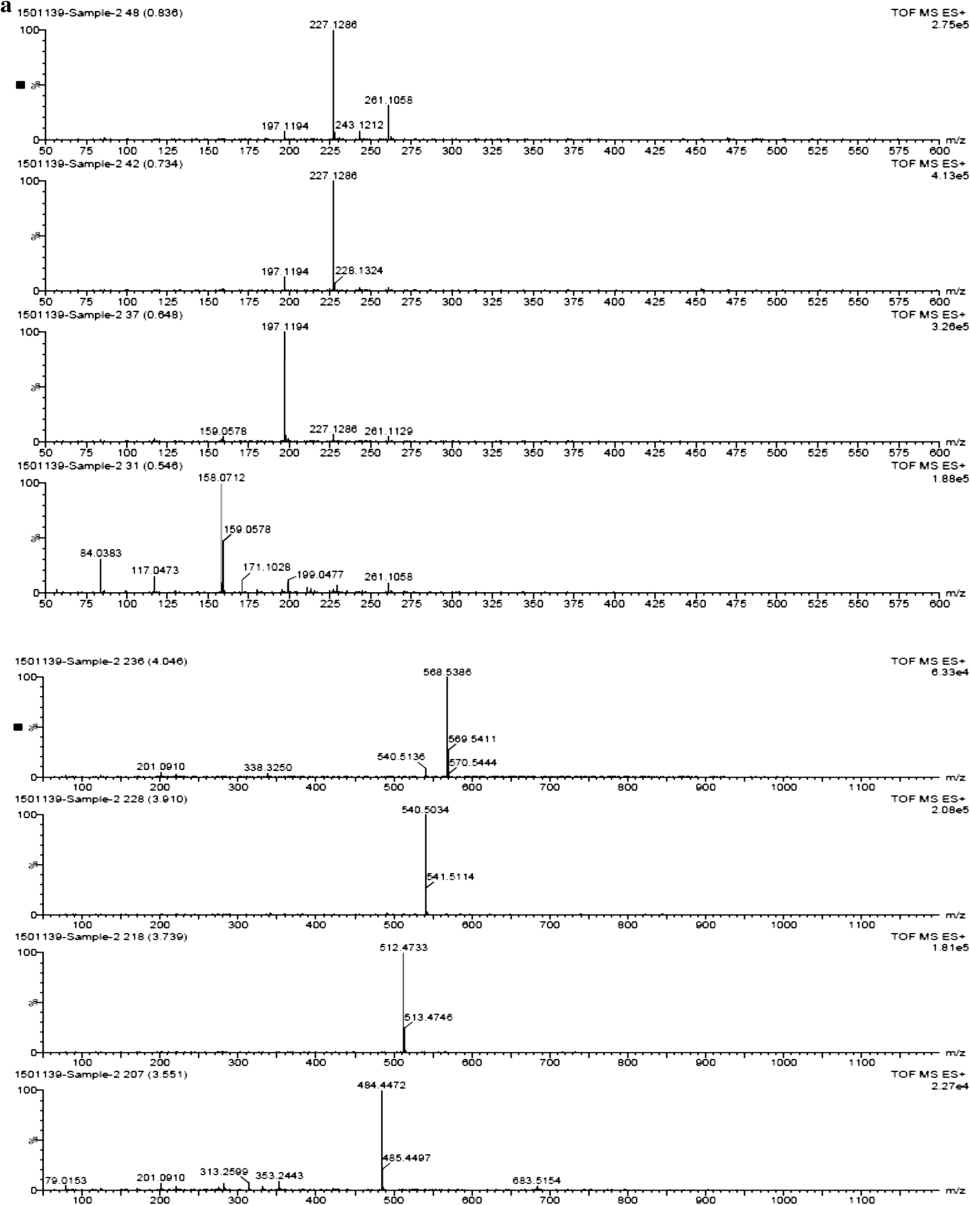

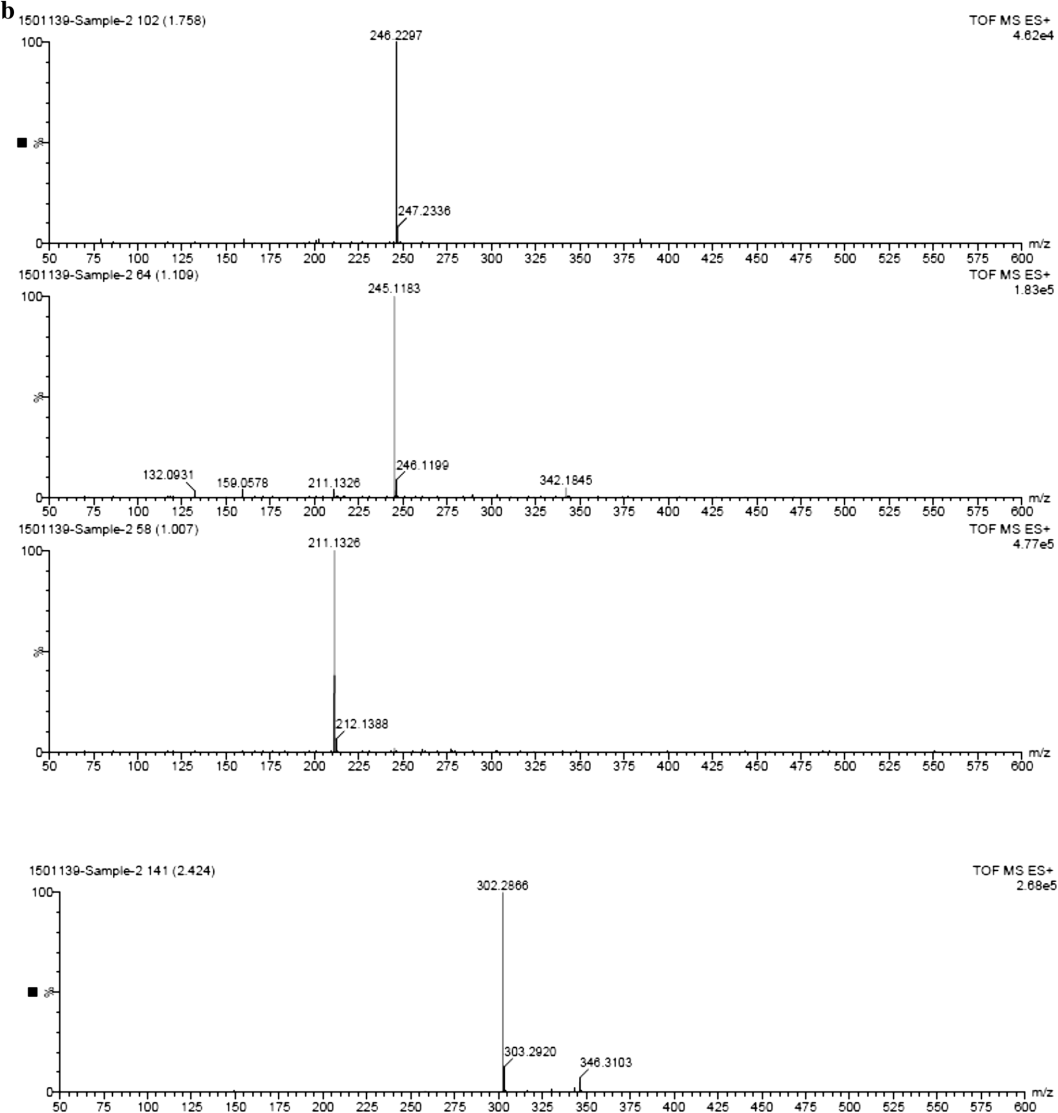

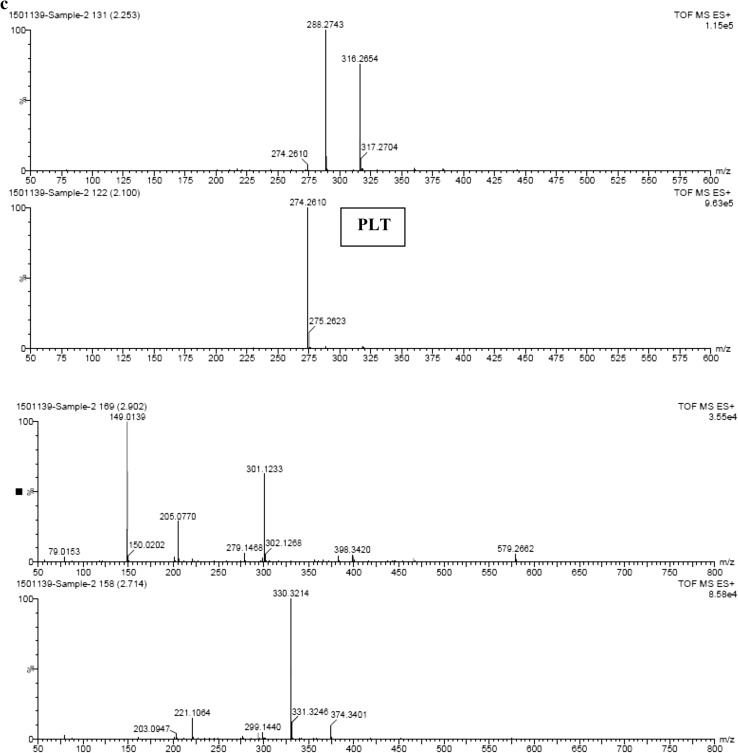
**a** TOF–MS profiling of *P. putida* RFP-4 extract showing different peaks. **b** TOF–MS profiling of *P. putida* RFP-4 extract showing different peaks. **c** TOF–MS analysis of pyoluteorin antibiotic (RFP-4) showing peak at 274.26 (g/mol)

**Fig. 3:**
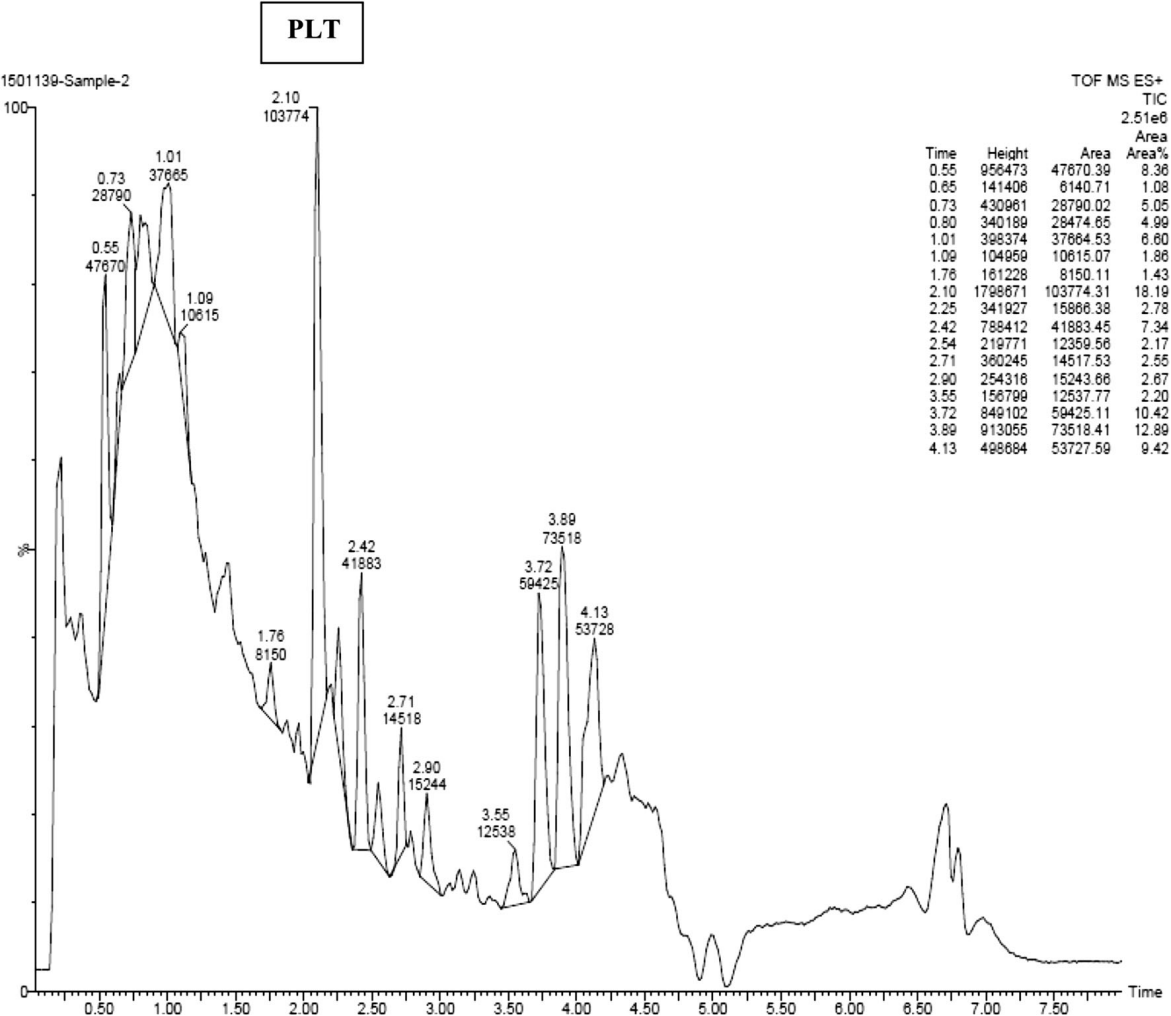
TOF–MS analysis of pyoluteorin antibiotic (RFP-4) showing retention time at 2.10 min

The pyoluteorin antibiotic extract of *P. putida* strain RFP-4 was analyzed through TOF–MS. The TOF–MS analysis of RFP-4 strain extract displayed 17 individual discrete chromatograms. These mass chromatograms showed 20 intense peaks at 227.12, 197.11, 158.07, 246.22, 245.11, 211.13, 302.28, 227.12, 288.27, 316.26, 274.26, 149.01, 301.12, 330.32, 304.28, 344.29, 568.53, 540.50, 512.47, and 484.44 (Fig. 2a, b). Many minor peaks also noticed in the chromatograms. In time-of-flight analysis, 17 different retention times 0.55, 0.65, 0.73, 0.80, 1.01, 1.09, 1.76, 2.10, 2.25, 2.42, 2.54, 2.71, 2.90, 3.55, 3.72, 3.89, and 4.13 min were obtained. Among different peaks and retention times, 274.26 (g/mol) peak (Fig. 2c) and 2.10 min retention time (Fig. 3) indicate the presence of pyoluteorin antibiotic in the RFP-4 strain’s antibiotic extract.

## Discussion

In the era of soil pollution and pesticide resistance in agriculture, there is need of better substitute for agro-chemicals. The next best choice was biological control in plant diseases. Therefore, necessity of selection of potential isolates with efficient secondary metabolites production was present target of many researcher’s in the field of biological control. Due to failure of many pesticides in plant disease control, secondary metabolites of biocontrol agents, such as fluorescent pseudomonads, were future eco-friendly weapons in targeting the plant diseases due to antimicrobial characteristics of those metabolites. Therefore, identifying and retrieving of antimicrobial traits from fluorescent pseudomonads were done in the present investigations.

As an initial step in the identification and exploitation of bioagents, collection and characterization of fluorescent pseudomonad isolates were done from diverse niche of their survival, i.e., from different crop rhizospheres in Raichur, and some more additional isolates were also collected from Chitradurga and Dharwad for the comparison of nature and performance among isolates. In addition, characterization of these isolates was done and the result of identification of these isolates was compared with identification and screening for pgpr traits (Laha and Verma 1998; Omolola 2007; Meera and Balabaskar 2012; Manjunath et al. 2011; Deshwal and Kumar 2013).

The antimicrobial nature of these secondary metabolites was confirmed by Fiddaman and Rossall (1993) who reported the fungal cell-wall degradation activity of HCN, which was produced from *Pseudomonas fluorescens.* Manidipa et al. (2013) confirmed that production of antibiotics, siderophores, volatile compounds, hydrocyanic acid (HCN), enzymes, and phytohormones in *Pseudomonas,* effectively controlled the fungal and bacterial diseases of rice.

Screening of 30 fluorescent pseudomonads against *R. solani*, gifted a nine fruitful superior strains RFP-3, RFP-6, RFP-7, RFP-10, RFP-19, RFP-20, RFP-21, RFP-22, and RFP-26 which were showing more than 40 % mycelial inhibition in dual culture technique.

The ten genes, *pltA, B, C, D, E, F, G, L, R,* and *M,* were required for the pyoluteorin biosynthesis by *Pseudomonas fluorescens* pf-5 (Nowak-Thompson et al. 1997). Each of these genes has their own pivotal role in biosynthesis of pyoluteorin (Nowak-Thompson et al. 1999). In the present investigation, *pltB* gene was detected using polymerase chain reaction (PCR) using the gene specific primers, pltBf and pltBr. Among 35 isolates of fluorescent pseudomonads, two isolates (RFP-4 and RFP-19) showed successful amplification at 779 bp region in the genome of these isolates. That region corresponds to *pltB* gene, which encodes the biosynthesis of pyoluteorin antibiotic. The results are in correspondence to those Saikia et al. (2011) who reported that, out of the 25 isolates, pyoluteorin (PLT) antibiotic-coding gene was detected in the isolate, Pf373. The 779 bp gene (pltB) amplification was earlier reported by Naik et al. (2008) and Ashwitha et al. (2013).

Further identification of species of pyoluteorin positive fluorescent pseudomonads through 16S rRNA sequence analysis revealed that the two pyoluteorin positive isolates RFP- 4 and RFP-19 belong to *Pseudomonas putida.* The RFP-4 strain was isolated from groundnut rhizosphere, KVK, Raichur, and RFP-19 strain was isolated from tomato, Chandraband, Raichur. The RFP-22 strain showed maximum zone of inhibition against *R. solani.* This strain belongs to *Pseudomonas putida* which was isolated from sesamum rhizosphere, MARS, Raichur.

Pyoluteorin antibiotic detection was done through TLC based on mobility of the compound on the silica plate that is measured in terms of retardation factor (*Rf* value). The *Rf* value of 0.50 corresponded to pyoluteorin antibiotic with chloroform: acetone solvent system and confirmed the expression of PLT genes as detected earlier. The pyoluteorin antibiotic detected at based on *Rf* value (0.50) by Cazorla et al. (2006), Ayyadurai et al. (2007) and Naik et al. (2008).

The TOF–MS characterization helped in identification of unknown metabolites in sample based on the peaks formed in chromatogram which was correspondent to molecular weight of that compound. In addition to that, retention time is also a key factor in identification of unknown compound. The TOF–MS analysis of RFP-4 strain antibiotic extract displayed 17 individual discrete chromatograms with different peaks and retention time. Among different peaks and retention time, 274.26 (g/mol) peak and 2.10 min retention time confirmed the presence of pyoluteorin antibiotic in the RFP-4 strain. These mass chromatograms showed 20 intense peaks, several minor peaks, and different retention time, and indicated the presence of unknown secondary metabolites and splitting of ions from parent molecules in the *P. putida* RFP-4 extracted solvent. Some of the metabolites also noticed at undetectable level, which were showed minute peak in chromatograms.

The characterization of pyoluteorin antibiotic through PCR, chromatography, and TOF–MS helped in selection of pyoluteorin positive strains of fluorescent pseudomonads for future successful disease management, although pyoluteorin antibiotic was most effective against oomycete plant pathogens (Howell and Stipanovic 1980). The present investigation gifted superior fruitful strains of fluorescent pseudomonads from different crop rhizospheres. Among these, two strains were proved as pyoluteorin (pltB) positive with different stages of confirmation of pyoluteorin antibiotic production, which are gene level through PCR and its product level through TLC and TOF–MS.
